# Debriefing Methodologies in Nursing Simulation: An Exploratory Study of the Italian Settings

**DOI:** 10.3390/nursrep15010007

**Published:** 2024-12-30

**Authors:** Sonia Lomuscio, Emanuele Capogna, Stefano Sironi, Marco Sguanci, Sara Morales Palomares, Giovanni Cangelosi, Gaetano Ferrara, Stefano Mancin, Antonio Amodeo, Anne Destrebecq, Mauro Parozzi, Susy Dal Bello

**Affiliations:** 1 Department of Biomedicine and Prevention, University of Rome Tor Vergata, 00133 Rome, Italy; sonia.lomuscio.01@students.uniroma2.eu (S.L.); mauro.parozzi@students.uniroma2.eu (M.P.); 2 EESOA, Simulation Center, 00151 Roma, Italy; capogna.eesoa@gmail.com; 3 IR&TeC-AREU e Centro di Simulazione, Agenzia Regionale Emergenza Urgenza, 20124 Milan, Italy; s.sironi@areu.lombardia.it; 4 A.O. Polyclinic San Martino Hospital, Largo R. Benzi 10, 16132 Genova, Italy; marco.sguanci@unicampus.it; 5 Department of Pharmacy, Health and Nutritional Sciences (DFSSN), University of Calabria, 87036 Rende, Italy; sara.morales@unical.it; 6 Unit of Diabetology, Asur Marche—Area Vasta 4 Fermo, 63900 Fermo, Italy; giovanni.cangelosi@virgilio.it; 7 Nephrology and Dialysis Unit, Ramazzini Hospital, 41012 Carpi, Italy; amaranto1984@libero.it; 8 IRCCS Humanitas Research Hospital, Via Manzoni 56, 20089 Rozzano, Italy; 9 Department of Oncological Urology, Veneto Institute of Oncology IOV—IRCCS, 35128 Padua, Italy; susy.dalbello@iov.veneto.it; 10 Department of Biomedical Science for Health, University of Milan, Via Pascal 36, 20133 Milan, Italy; anne.destrebecq@unimi.it

**Keywords:** nursing, simulation, debriefing, cross-sectional study, education

## Abstract

**Background:** As part of simulation-based learning, it is well known that debriefing plays a crucial role; ineffective debriefing can lead to a reiteration of errors in decision-making and a poor understanding of one’s limitations, compromising the learner’s psychological safety and making future simulated learning experiences less effective. In Italy, although simulation has been used in nursing education for more than 20 years, there is a general lack of data regarding the elements of debriefing. **Methods:** An exploratory, cross-sectional, multicenter nationwide study was conducted to identify current debriefing practices in Italian simulation-based nursing education. A non-probability sample of all directors of the Italian Bachelor school of Nursing and the directors of simulation centers on Italian national territory was surveyed with an online questionnaire. **Results:** Fifty-four nursing degree programs and 11 simulation centers participated in the survey. Significant differences were found between debriefing practices used by simulation centers and those used by the Bachelor School of Nursing. Specifically, differences concerned the training of debriefers, the knowledge of a debriefing framework, the use of different rooms for debriefing and the time spent on this activity. **Conclusions:** There is an emerging need for a harmonization process in Italian nursing education debriefing practices that would align the current reality with the best practices of the literature.

## 1. Introduction

The use of simulation as a learning strategy is, to date, a well-established methodology of training in health care [1,2,3,4], effective in both the development of technical and procedural skills [1,5,6,7,8,9] as well as behavioral and interpersonal skills [7,10,11,12,13], proving superior to other learning methods in basic and post-basic training [14,15].

The literature suggests that the critical moment of learning through simulation is debriefing [16,17,18,19]. This activity takes the form of the careful, engaged and purposeful reflection conducted by an experienced trainer who can stimulate participants’ self-analysis, through facilitation, to conceptualize the experience by recognizing the occurred events through the interpretation of meaning, improving the outcome [20,21,22,23,24,25,26]. The importance of reflection to consolidate learning after an experience is embedded in the theories of educational pioneers such as Dewey, Lewin, Piaget and Kolb [27]; by developing self-reflection through debriefing [28], learners acquire knowledge [29], skills [30], teamwork and communication skills [31], increasing self-confidence and satisfaction within their professional profile [32,33].

On the other hand, ineffective debriefing can also lead to a reiteration of errors in decision-making and a poor understanding of one’s limitations [34], compromising psychological safety [35,36] and making future simulated learning experiences less effective [37]. Given these premises, specific training for debriefing facilitators should become a requirement [13,38].

The International Nursing Association for Clinical Simulation and Learning (INACSL) published evidence-based guidelines for debriefing in 2016 [13], indicating some of the essential elements of the debriefing process [13,18]:

1. The expertise of the debriefer;
2. The elements of the debriefing environment;
3. The debriefing methodology used;
4. The use of objectives to guide the discussion.

The publication of these guidelines made it necessary to map the status of congruence and appropriateness of debriefing processes used in nursing education where simulation learning is widely used for basic education [18,24,32,39] to support and even replace clinical learning [18,40,41].

Globally, a limited number of studies have thoroughly investigated which elements of debriefing are actually used in nursing education within the Bachelor School of Nursing (BSN) and simulation centers (SC). Among them, one study [18] developed an advanced tool to describe the elements of debriefing in simulation in 1440 accredited nursing education programs in the United States. The same tool was used in South Korea [42], involving 95 nursing education centers using simulation.

In Italy, although learning by simulation has been a teaching methodology used in nursing education for more than 20 years, there is a general lack of data regarding the elements of debriefing [7,43,44]. Therefore, in order to probe the state of the art, it is necessary to use a tool that can compare the Italian reality with the INACLS standards regarding debriefing in nursing education through simulation [20,21,22,23,24,25,26].

The aim of this study, therefore, was to assess whether the tool designed by Fey [18], suitably modified for the Italian context, could be a valuable tool to map the Italian reality on current debriefing practices in both nursing education and simulation centers by conducting a preliminary mapping.

## 2. Materials and Methods

### 2.1. Study Design

A exploratory, cross-sectional, multicenter nationwide Italian study with preliminary validation of an instrument designed to identify current debriefing practices in simulation-based nursing education was conducted.

### 2.2. Sample

A non-probability sample consisting of all directors of three-year nursing degree programs and the directors of simulation centers on Italian national territory was used. In June and July 2023, a formal request for participation in this study was sent via e-mail to all subjects of interest, including the link to the instrument used for the survey. Participation in the study was voluntary.

### 2.3. The Questionnaire

The tool used for this study was from Fey’s Final Survey Questions: Debriefing Practices [18], translated into Italian using the methodology of back translation [45,46] after obtaining the author’s permission and appropriately modifying it for local cultural setting. The Italian-translated version of the instrument was preliminarily verified by eight experts with >5 years of experience in simulation. Of the forty-one questions in the four sections of the original instrument, five were evaluated by the experts as inappropriate for the Italian context and were therefore excluded (n. 3, 4, 9, 19, 20); the remaining 36 questions were culturally readjusted and subsequently used in this study. This study’s source definitions were taken from the *Dictionary of Medical Simulation* [47], which was translated into Italian by SIMMED (Italian Society of Simulation in Medicine) so that a shared language could be used. The final version of the questionnaire was divided into four different sections:

SECTION 1: Characteristics of the Graduate Course/Simulation Center (n.6 questions);

SECTION 2: Characteristics of the simulation expert (n.10 questions);

SECTION 3: Elements of debriefing (n.17 questions);

SECTION 4: Characteristics of the debriefer (n.3 questions);

The questionnaire, transposed online through the Google Forms platform, was subjected to face validity [48] by a panel of 10 experts (8 professors from the Bachelor of Science in nursing degree programs and 2 Directors from Simulation Centers), immediately obtaining an overall and single item % of agreement >90%. The instrument was subsequently subjected to content validity [49] by the same panel of experts with satisfactory results (I-CVI = always > 0.80).

In order to assess its reliability and preliminary validity, the instrument was then subjected to the calculation of Cronbach’s alpha, McDonald’s omega and a correlation matrix performed through the Jamovi software (V. 2.3.21).

### 2.4. Data Collection

Conducting the data collection face-to-face by personally administering the questionnaire was not possible. Due to the territory to be surveyed and limited staff, an online (not face-to-face) survey was conducted. The official email addresses of the directors of the Degree Courses in Nursing on Italian territory were, therefore, retrieved by requesting access to the Permanent Conference of the Degree Classes of Health Professions (the Italian national body that coordinates the development of the educational activities of the Degree Courses in health care); likewise, the official email addresses of the directors of the simulation centers were retrieved by officially requesting access to the Italian Society of Simulation in Medicine (SIMMED). The emails of the study participants were not collected at the compilation stage, keeping the responses to the questionnaire anonymous. The data were subsequently handled separately, dividing the responses between those from the nursing degree courses and those from the simulation centers.

### 2.5. Data Analysis

The collected data were analyzed through the Jamovi software (V. 2.3.21). Characteristics of degree courses and simulation centers were analyzed through descriptive statistics. Missing data were processed using the pairwise deletion methodology, which involves using only the available data for each analysis. The association between the obtained responses and membership in a degree program versus a simulation center was assessed using the Chi-square test with Yates correction (40 < N < 200).

### 2.6. Authorizations

The director of the nursing degree course of the University of Milan approved the first conduction of this study in 6 October 2022. The study was discussed in the session of 21 January 2023 and authorized by the Institutional Review Board of the Bachelor School of Nursing of the University of Milan—ASST Lodi, with registration number N. SL/SM003.

## 3. Results

### 3.1. Reliability Analysis

Reliability analysis should have been conducted separately between SC and BSN. However, since the number of SC that joined the study turned out to be small (N. 11 centers out of 34 total), reliability analysis was conducted on BSN data only. In addition, items 1, 2, 7, 15, 18 and 32 were excluded from the analyses because they aimed to gather additional qualitative information concerning the preliminary mapping of debriefing elements on the national territory.

Cronbach’s α and McDonald’s Ω coefficients [50] were calculated globally and with the subtraction of individual items. Both coefficients were at globally acceptable values [51,52] (α = 0.799, Ω = 0.749), indicating some internal consistency in the instrument used. The exclusion of individual items produced no notable changes.

Next, given how the literature [53,54] indicates the absence of a sufficient number of responses to perform an exploratory factor analysis (EFA), an items correlation matrix was analyzed (available at https://doi.org/10.6084/m9.figshare.25003790, accessed on 20 January 2024). Assuming a priori, a non-normal distribution due to the smallness of the sample, Spearman’s rho test was used to assess the significance of the correlation between the items. The existence of several significant correlations among the items was thus verified, which will need to be further investigated by factor analysis in the presence of a larger sample size. Finally, Bartlett’s sphericity test assessed any differences with an identity matrix. The test was significantly affirmed for values < 0.001 (χ^2^ = 860, gdl = 435), rejecting the null hypothesis. Thus, the instrument used demonstrated psychometric characteristics of internal consistency and reliability, outlining a promising construct that needs further exploration and confirmation in the presence of additional data.

### 3.2. Questionnaire Results

#### 3.2.1. Bachelor Courses in Nursing

A total of 54 bachelor’s nursing schools out of the 211 invited respondents (response rate = 25.6%) responded to the questionnaire. The questionnaire collected 54 responses, of which only 42 were fully completed. The bachelor’s degree programs that responded correctly are located in 13 of the 20 Italian regions, with a relative majority of responses coming from bachelor’s degree programs located in Northern Italy (n = 22, 47.8%) followed by Central Italy (n = 14, 36.8%), Southern Italy (n = 6, 13%) and the islands (n = 1, 2.2%). The number of degree course enrollees who responded proved highly variable, with a mean of 262, ranging from 22 to 1150 students. Simulation experts in graduate courses were aged between 36 and 45 years (n = 16, 34.8%) with a master’s degree in nursing science (n = 28, 64%) and have mainly been involved in simulation for 1 to 3 years (n = 21, 44%).

#### 3.2.2. Simulation Centers

Eleven simulation centers out of the thirty-four invited responded to the questionnaire (response rate: 32.3%), with 63% (n = 7) from Northern Italy, 18% (n = 2) from Central Italy and 9% (n = 1) from Southern Italy (missing data, n = 1). These centers reported receiving a mean of 2792 students annually with a range of 50 to 12,000 students. Simulation experts in training centers were mainly aged between 36 and 45 years old (n = 4, 57%), and the highest academic qualification held is a master’s degree (n = 6, 75%); furthermore, the simulation centers also specified that their staff belonged to different professional profiles: physician, nurses, paramedics and psychologists. A total of 81.8% have been using simulation as a teaching methodology for more than 3 years. Table 1 summarizes the described results.

All SC (100%) vs. 81.5% of responding BSN identified the definition of simulation as that taken from the *Dictionary of Simulation in Medicine* [47] translated by SIMMED. The presence of a facilitator (as defined by the same dictionary, while standing at 81.8% in SC vs. 40.7% (with N = 46) of BSN was not significant (χ^2^(Yates) = 1.167, *p*= 0.28).

### 3.3. Characteristics of the Designated Expert

There is a higher percentage of experts with formal recognition in SCs (45.5%) than in BSN (n = 9/46), which, however, does not reach statistical significance (χ^2^(Yates) = 1.614, *p* = 0.203). In the two settings, an inverse trend is denoted concerning the workload devoted to the simulation of the simulation expert; in degree courses, the majority devote 25% or less of their working time to simulation, while the majority of experts in simulation centers (45.5%) would appear to devote between 76 and 100% of their working time to this activity (χ^2^(Yates) = 12.104, *p* < 0.001). Center experts overwhelmingly (91% of cases) report having received specific training compared with 50% (with n = 21/42) of the simulation subjects in nursing degree programs; this difference was found to be statistically significant (χ^2^(Yates) = 4.442, *p* = 0.035). Likewise, simulation experts belonging to simulation centers overwhelmingly state that they are members of organizations concerning simulation or are accredited for such activities (54.5%); in nursing degree programs, this is the case for only 21.7% of experts (n = 10/46). This difference between the two settings was found to be statistically significant with the Chi-square statistic (χ^2^= 4.731, *p* = 0.029, N = 56) but not with the Yates-corrected test (χ^2^(Yates) = 3.246, *p* = 0.071, N = 56). Table 2 summarizes the data described.

#### Current Pre-Briefing and Debriefing Practices

Table 3 describes the results of pre-briefing and debriefing practices within the two simulation settings studied. Among the variables investigated through Fey’s modified questionnaire concerning pre-briefing, individual comparisons made between nursing degree programs and simulation centers regarding discussion of simulation limitations (χ^2^(Yates) = 6.19, *p* = 0.013), sham contract (χ^2^(Yates) = 6.0, *p* = 0.014) and whether the simulation will be recorded with (χ^2^(Yates) = 8.85, *p* = 0.003) were statistically significant.

Experts were asked whether they knew of a theoretical framework to guide them in debriefing; A total of 81.9% of simulation centers answered in the affirmative, while the percentage reported by nursing degree programs was lower (38.8%). Membership in a simulation center was significant with χ^2^(Yates) = 5.07, *p* = 0.0024 (N = 60). The theoretical frameworks known by both settings were Plus-Delta debriefing (which was found to be the most well-known), strategic debriefing, 3d model debriefing and GAS. GAS was found to be the most widely applied model in practice in both settings, followed by strategic debriefing. CSs also reported the use of 3d model debriefing. Membership in a simulation center and using assessment models for debriefing were significantly associated (χ^2^ = 10.156, *p* = 0.001).

Belonging to a BSN was significantly associated with holding the debriefing in the same room where the simulation is conducted (χ^2^(Yates) = 6.62, *p* = 0.01, N = 56); likewise, belonging to a SC was associated with holding the debriefing in a separate room from the simulation room (χ^2^(Yates) = 6.62, *p* = 0.01, N = 56).

All simulation centers (100%) reported that debriefing is conducted immediately after the simulation; in BSN, this percentage is lower (67.3%). Membership in a BSN was significantly associated with debriefing times of 50% or less of the time spent in simulation (χ^2^(Yates) = 5.90, *p* = 0.015). In contrast, membership in a simulation center was significantly associated with debriefing times of about twice the time used for simulation (χ^2^(Yates) = 17.45, *p*= < 0.001).

The evidence found is further discussed below.

### 3.4. Characteristics of the Debriefer

While the majority of SCs (63.6%) reported that the debriefer possesses advanced simulation training, in BSN, this percentage appears to be lower (23.4%); the association found between SC membership and advanced training of the debriefer was found to be statistically significant (χ^2^(Yates) = 4.992 *p* = 0.025). This training was found to be provided only as a SC-specific training. In BSN, training is also carried out among peers.

In both settings, debriefer performance evaluation is still underutilized (SC = 27.3%, BSN = 21.3%); moreover, when carried out, it would appear that only a narrow minority resort to the use of validated instruments already found in the literature.

## 4. Discussion

This study conducted a preliminary mapping of debriefing practices in the Italian national context by culturally repurposing a questionnaire already used in the literature [18] into an Italian context. Reliability analysis found good values for both Cronbach’s α and McDonald’s Ω [49]. The limited sample, which did not allow for exploratory factor analysis, allowed for significant correlations between items in the correlation matrix. These results seem to indicate a good reliability of the instrument used, which will have to be confirmed in the future against a larger sample.

To the best of our knowledge, only a few studies [18,42] in the literature have similarly mapped debriefing practices, but none of them specifically compares simulation practices of BSN with those of SC.

The significant differences that emerged between the two settings seem to properly reflect the Italian national reality where learning by simulation has only been recently introduced and normalized in BSN in nursing [43], while in the SCs, there is a more pronounced alignment with international standards [13] on pre-briefing and debriefing practices.

The lack of specific training for those who facilitate debriefing at BSN and the more specific training for those who facilitate debriefing at SC aligns with the findings of Fey’s [18] and Kim’s [42] studies, respectively. A likely explanation for this trend could lie in the broader variability of activities performed within a BSN compared to a SC, leading SC facilitators to conduct more specific training on the topic.

The specific training of the instructors working within the SC and the greater amount of time they state they devote to debriefing could also explain a greater focus on some aspects of pre-briefing defined as essential in the literature such as explaining the role of the debriefer, the peculiarities that the pretest contract must possess, including the limits of the simulation, and taking care of the psychological safety of the learner [35,36]. These characteristics could also explain the greater theoretical knowledge of debriefing models and their use. Interestingly, the GAS model, which emerges in the literature as one of the most easily applied models, is reported as the most widely used debriefing model by both SC and BSN.

While SC are structurally built to have places set up for both simulation activities and the time of debriefing, BSNs are often located within hospital facilities that are built for different purposes, where there are limitations in space management; this is probably one of the reasons that helps explain why, within BSN, debriefing is mainly carried out in the same simulation environment, not aligning with the evidence in the literature that indicates an external, separate simulation environment as the first choice. On the other hand, SC practice was found to be aligned with this evidence, as was noted in Kim’s study [42].

As can be seen from the results, the amount of time spent on simulation activities in BSN is significantly less than that in SC. This element may partly explain why debriefing is not always performed after simulation [19,55] and why the duration of debriefing carried out in BSN is significantly less than that stated by SC. Indeed, in BSN, learning by simulation, when present, constitutes only a limited part of the training activities to be carried out by instructors; therefore, the time devoted to this kind of activity may not always be adequate.

The existence of differences between SCs and BSNs in the various elements of debriefing, such as those found by this study, must necessarily cast doubt on the actual effectiveness of the training received in sub-optimal settings or with practices not aligned with INACSL standards. Our data do not allow us to define the degree of effectiveness of one over the other; however, it would seem safe to assume that nursing students who have the opportunity to attend a simulation center receive more effective training.

To date, studies have emerged on how poorly performed debriefing can negatively affect the learning experience [13] and the importance of evaluating debriefers in their respective activities. However, in both settings, debriefer performance assessment still appears to be underutilized; future investigations may clarify whether this finding reflects only a relatively recent implementation of validated debriefer performance assessment tools or an ongoing trend that should be reversed.

A harmonization process in debriefing practices should first begin with the establishment of nationally recognized training pathways for simulation facilitators and debriefers; subsequently, technical commissions to analyze and overcome existing critical issues with a shared approach aimed at maximizing student learning outcomes with the available resources in the Italian educational context need to be established. In this situation, the Italian National Federation of Nursing Profession Orders (FNOPI) could have a pivotal role in coordinating and supervising.

### Limitations

Considering that we wanted to conduct a preliminary mapping of the whole country with limited resources, the online mode of administration probably affected the response rate to the questionnaire, which was limited, especially in the context of the SC. In addition, the questionnaire was structured in an online format with many items, which may have affected the completeness of responses. The sample selection was also non-probabilistic, which may limit the representativeness of the data. The observed correlations between items should be interpreted cautiously due to the small sample size.

## 5. Conclusions

Significant differences concerning the elements of debriefing within the settings analyzed and in comparison with the standards outlined in inter-national guidelines emerged. It would be interesting to understand whether these differences are attributable to the recent introduction of simulation activities in BSN, to the need for more specific training or to the need for more time to devote to simulation activities. However, regardless of the reasons, there is an emerging need for a harmonization process in debriefing practices that would align the current reality with the best practices of the literature.

## Figures and Tables

**Table 1:** Characteristics of various bachelor schools of nursing and simulation centers.

	Setting	
	BSN	SC
Geographical setting:		
N. (%)	46 (100%)	10 (100%)
Northern Italy	22 (47.8%)	7 (63%)
Center Italy	14 (36.8%)	2 (18%)
South Italy	6 (13%)	1 (9%)
Island	1 (2.2%)	0 (0%)
Students/Year:		
N. (%)	46 (100%)	9 (100%)
Range (min-max)	22–1150	50–12,000
IQ Range:	98.5–300	900–2000
Mean	262	2792
Median	186	1100
Assignment of the simulation expert’s role		
N. (%)	46 (100%)	11(100%)
Formal	9 (20%)	5
Informal	34 (74%)	6
There is no Expert	3 (6%)	0 (0%)
Simulation expert age (Years old)		
N. (%)	45 (100%)	7 (100%)
<25	1 (2%)	0 (0%)
26–35	8 (18%)	0 (0%)
36–45	16 (36%)	4 (57%)
46–55	13 (29%)	2 (29%)
>56	4 (9%)	1 (14%)
There is no Expert	3 (7%)	0 (0%)
Simulation expert qualification		
N. (%)	44 (100%)	8 (100%)
Bachelor’s degree	9 (20%)	2 (25%)
Master’s degree	28 (64%)	6 (75%)
PhD	4 (9%)	0 (0%)
There is no expert	3 (7%)	0 (0%)
Expert use of simulation to teach		
N. (%)	48 (100%)	10 (100%)
1–3 years	21 (44%)	2 (20%)
4–6 years	10 (21%)	3 (30%)
7–10 years	4 (8%)	2 (20%)
≥11 years	8 (17%)	3 (30%)
There is no expert	5 (10%)	(0%)

Legend: BSN: bachelor of science in nursing; SC: simulation center; IQ: interquartile; N.: number; %: percentage; Min: minimum; Max: maximum; PhD: Doctor of Philosophy.

**Table 2:** Designed simulation expert characteristics.

Bachelor Schools of Nursing						
Type of assignment	Formal	Informal	Other			
n (%)	9 (19.6)	34 (73.9)	3 (6.5)			
Workload over total	25% or less	26–50%	51–75%	76–100%	Other	
n (%)	28 (60.9)	9 (19.6)	5 (10.9)	1 (2.2)	3 (6.5)	
Years of teaching at work	1–5	6–10	11–15	16–20	21 or more	Other
n (%)	20 (43.5)	10 (21.7)	9 (19.6)	1 (2.2)	3 (6.5)	3 (6.5)
Teaching years with simulation	1–3	4–6	7–10	11 o più	Other	
n (%)	21 (45.7)	10 (21.7)	4 (8.7)	8 (17.4)	3 (6.5)	
Expert Age	<25	26–35	36–45	46–55	56 or more	Other
n (%)	1 (2.2)	8 (17.4)	16 (34.8)	4 (8.7)	4 (8.7)	13 (28.3)
Highest owned academic degree	Bachelor	Master	Ph.D.	Other	Other	
n (%)	8 (17.4)	28 (60.9)	4 (8.7)	1 (2.2)	5 (10.9)	
Expert formal training in simulation	Yes	No	Other			
n (%)	21 (46.7)	21 (46.7)	4 (8.7)			
Member of organizations or registered for simulation	Yes	No				
n (%)	10 (21.7)	36 (78,3)				
**Simulation Centers**						
Type of assignment	Formal	Informal				
n (%)	5 (45.5)	6 (54.5)				
Workload over total	25% or less	26–50%	51–75%	76–100%		
n (%)	2 (18.2)	1 (9.1)	3 (27.3)	5 (45.5)		
Years of teaching at work	1–5	6–10	11–15	16–20	21 or more	
n (%)	7 (63.6)	1 (9.1)	2 (18.2)	0 (0)	1 (9.1)	
Teaching years with simulation	1–3	4–6	7–10	11 or more	Other	
n (%)	2 (18.2)	3 (27.3)	2 (18.2)	3 (27.3)	1 (9.1)	
Expert Age	<25	26–35	36–45	46–55	56 or more	Other
n (%)	0 (0)	0 (0)	4 (36.4)	2 (18.2)	1 (9.1)	4 (36.4)
Highest owned academic degree	Bachelor	Master	Ph.D.	Other	Other	
n (%)	4 (36.4)	1 (9.1)	0	1 (9.1)	5 (45.5)	
Expert formal training in simulation	Yes	No	Other			
n (%)	10 (90.9)	1 (9.1)	-			
Member of organizations or registered for simulation	Yes	No	Other			
n (%)	6 (54.5)	5 (45.5)	-			

Legend: BSN: bachelor schools of nursing; SCs: simulation centers; n.: number; %: percentage; Min: minimum; Max: maximum; PhD: Doctor of Philosophy; Other: not specified or alternative category.

**Table 3:** Debriefing items.

Variable	Division	Bachelor of Nursing	Simulation Centers
		n. (%)	n. (%)
Pre-briefing activity	Definition and learning goals of simulation education	38 (76)	10 (90.9)
	Understand learners’ thinking	28 (56)	7 (63.6)
	Lecturers’ role during debriefing	29 (58)	11 (100)
	Time for simulation in progress	37 (74)	10 (90.9)
	Time for debriefing in progress	26 (52)	6 (54.5)
	Confidentiality of discussion during debriefing	24 (48)	8 (72.7)
	Students can experience emotional reactions	24 (48)	8 (72.7)
	Discussion on the limits of the simulator	22 (44)	10 (90.9)
	Simulation is similar to reality	14 (28)	8 (72.7)
	Recording of simulation	18 (36)	10 (90.9)
	Debriefing focused on students’ reflection	29 (58)	7 (63.6)
	Understanding of learners’ mistakes as an opportunity	40 (80)	10 (90.9)
	Cannot answer	2 (4%)	3 (27.2)
Knowledge of a guide model to debriefing	Yes	19 (38.8)	9 (81.8%)
	No	30 (61.2)	2 (18.2)
Debriefing guide model	3d model debfiefing	1	1
	Strategic debriefing	1	3
	Plus-Delta	4	5
	GAS	1	1
	Gibbs reflective’s circle	4	0
	Standard INACSL	1	0
	Lederman’s model	1	0
	NMDS	1	0
	PEARLS	3	0
	Zigmont	0	2
	Advocacy inquiring	0	3
	Good judgment	0	1
	Case review	0	1
Applying evaluation model during debriefing	Yes	16 (32.7)	10 (90.9)
	No	33 (67.3)	1 (9.1)
Applied model of debriefing	Strategic debriefing	1	1
	GAS	3	3
	Gibbs reflective’s circle	1	0
	ERA	1	0
	Plus-Delta	1	0
	PEARLS	1	0
	CRM	2	0
	3d model	0	1
Location of debriefing	In the simulation room	30 (61.2)	2 (18.2)
	Separated debriefing room	15 (30.6)	9 (81.8)
	Cannot answer	4 (8.2)	-
Time for debriefing	During the simulation’s experience	10 (20.4)	0 (0)
	Immediately after simulation of each group	33 (67.3)	11 (100)
	Some hours later after the simulation experience but on the same day	2 (4.1)	0 (0)
	After the simulation experience, during the following class period	0 (0)	0 (0)
	Debriefing does not follow most simulation experiences	1 (2.0)	0 (0)
	Cannot answer	3 (6.1)	0 (0)
Debriefing environment	Same simulation room	28 (57.1)	0 (0)
	Place separated from simulation room where chairs are arranged in a row	2 (4.1)	0 (0)
	Students are at a place separated from the simulation; students and instructor are separated at the same table.	3 (6.1)	0 (0)
	Space separated from simulation room where students and instructor sit together	19 (38.8)	10 (90.9)
	Instructor explains points, makes conceptual map and uses a white board to record	17 (34.7)	6 (54.5)
	The facilitator rewatches the video with the students during the debriefing	14 (28.6)	5 (45.5)
	During simulation, they are instructed in the debriefing to refer to assessment instruments used to record event and reaction	9 (18.4)	1 (9.1)
	Instructor who can affect the learner’s performance	0 (0)	1 (9.1)
	Cannot answer	4 (8.2)	0 (0)
Time spent on debriefing	50% or less of the simulation time	27 (55.1)	1 (9.1)
	More than 50% of the simulation time	5 (10.2)	0 (0)
	The same as the time for simulation	6 (12.2)	0 (0)
	About double the time for simulation	3 (6.2)	7 (63.7)
	Cannot answer	6 (12.2)	1 (9.1)
	Others	2 (4.1)	1 (9.1)

Legend: BSN: bachelor schools of nursing; SCs: simulation centers; n.: number; %: percentage; 3D: three-dimensional model; GAS: group assessment strategy; ERA: experience, reflection and action; CRM: crisis resource management; INACSL: International Nursing Association for Clinical Simulation and Learning; NMDS: National Model for Debriefing Standards; PEARLS: Promoting Excellence and Reflective Learning in Simulation.

## Data Availability

The data presented in this study are available upon reasonable request to the corresponding author.

## Public Involvement Statement

No public involvement in any aspect of this research.

## Guidelines and Standards Statement

This manuscript was drafted according to the Strengthening the Reporting of Observational Studies in Epidemiology (STROBE) Statement for cross-sectional studies.
